# Computational Analysis of the Nicotine Oxidoreductase Mechanism by the ONIOM Method

**DOI:** 10.1021/acsomega.1c03357

**Published:** 2021-08-18

**Authors:** Ibrahim Yildiz

**Affiliations:** Chemistry Department, Khalifa University, P.O. Box 127788 Abu Dhabi, United Arab Emirates

## Abstract

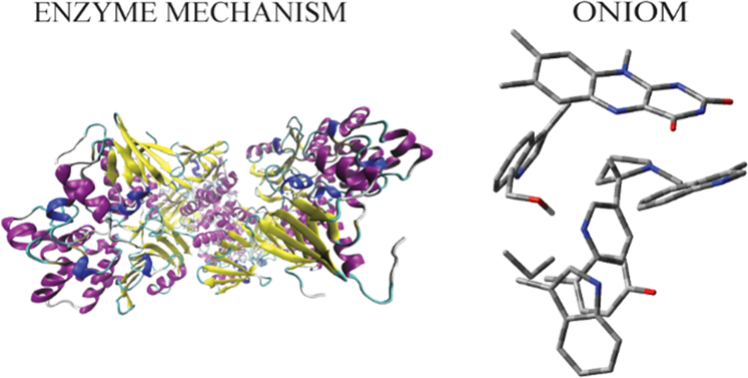

Nicotine oxidoreductase (NicA2) is a monoamine oxidase (MAO)-based flavoenzyme that catalyzes the oxidation of S-nicotine into *N*-methylmyosmine. Due to its nanomolar binding affinity toward nicotine, it is seen as an ideal candidate for the treatment of nicotine addiction. Based on the crystal structure of the substrate-bound enzyme, hydrophobic interactions mainly govern the binding of the substrate in the active site through Trp108, Trp364, Trp427, and Leu217 residues. In addition, Tyr308 forms H-bonding with the pyridyl nitrogen of the substrate. Experimental and computational studies support the hydride transfer mechanism for MAO-based enzymes. In this mechanism, a hydride ion transfers from the substrate to the flavin cofactor. In this study, computational models involving the ONIOM method were formulated to study the hydride transfer mechanism based on the crystal structure of the enzyme–substrate complex. The geometry and energetics of the hydride transfer mechanism were analyzed, and the roles of active site residues were highlighted.

## 1 Introduction

**Figure 1:**
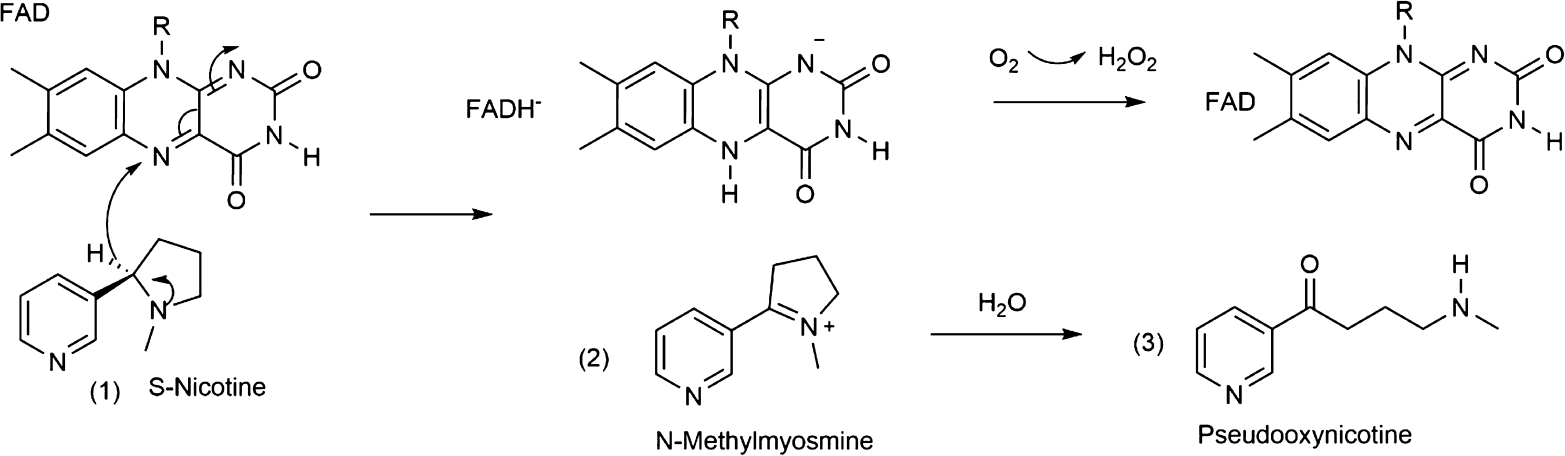
Oxidation of S-nicotine (1) into pseudonicotine (3).

Nicotine oxidoreductase (NicA2) was identified as the primary enzyme in the S16 bacterium that degrades S-nicotine into fumaric acid in a number of steps.^1^ Based on the crystal structures of the free and substrate-bound enzymes, the enzyme exists as a monomer consisting of a substrate and FAD-binding domains.^2,3^ From collective experimental and computational studies for monoamine oxidase (MAO) enzymes,^4−8^ in particular for L-6-hydroxynicotine oxidase (LHNO)^9−12^—a flavoenzyme structurally related to NicA2—, the direct hydride transfer mechanism from deprotonated S-nicotine to FAD is proposed for NicA2.^2,3^ Based on this mechanism, a hydride anion is transferred from the α-carbon of S-nicotine (**1** in Figure 1) to the N5 nitrogen of the isoalloxazine ring at FAD, forming reduced FAD (FADH^–^) and an iminium intermediate, *N*-methylmyosmine (**2**Figure 1). In a subsequent step, *N*-methylmyosmine hydrolyzes into pseudooxynicotine (**3** in Figure 1) by a water molecule nonenzymatically.

**Figure 2:**
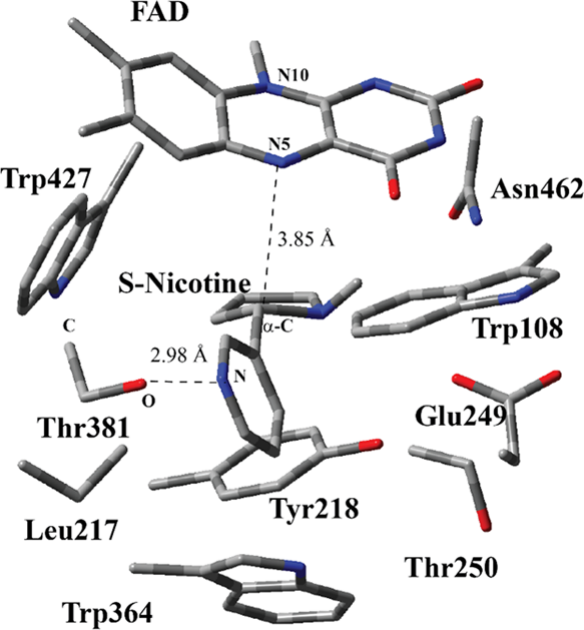
Structure of the active site of NicA2 complexed with S-nicotine, including surrounding residues and FAD.

The crystal structure of NicA2 complexed with S-nicotine shows that the substrate, S-nicotine, is surrounded closely by Trp108, Trp364, Trp427, Thr281, Leu217, and Tyr218^3^ (Figure 2). Four aromatic residues form a cage around S-nicotine, while O in Tyr 218 has close H-bonding interactions with the pyridyl N of S-nicotine. The pyrrolidine ring at S-nicotine is located just under the isoalloxazine ring of FAD. The α-carbon of S-nicotine is 3.85 Å away from the N5 position of the isoalloxazine ring. These observations are in agreement with those of other MAO family members and suggest a similar oxidation mechanism and mode of substrate binding.^10^ The isoalloxazine ring is bent about the N5–N10 axis, which is a common observation for the MAO family due to substrate binding and/or steric constraints around FAD.^12,13^

Mechanistic interpretations of the MAO family obtained from experimental and computational studies offer new opportunities for the design of therapeutics against neurodegenerative diseases.^14^ Furthermore, studies related to nicotine oxidases can provide novel strategies to deal with nicotine addiction and poisoning. Moreover, nicotine remediation could be performed to obtain organic molecules with synthetic importance.^15^ The hydride transfer mechanism is supported for a number of MAO family enzymes by experimental and computational studies.^4,16−24^ Kinetics and mutagenesis studies of NiAc2 have provided mechanistic information for the oxidation of S-nicotine.^3,25^ It was observed that *k*_cat_ is independent of pH, indicating that the deprotonated form of amine is not required for binding and catalysis.^3^ However, the hydrophobic environment around the substrate implies that the binding of the deprotonated form is more favorable. Enzyme activities using a panel of substrate analogues revealed that H-bonding involving the pyridyl N of S-nicotine affects the catalysis to a significant extent. NiAc2 has a very high affinity toward S-nicotine with a very low *K*_m_ value.^1^ However, the catalytic rate, 0.006 s^–1^, was very low when O_2_ was used as the oxidizing agent for the reduced FAD.^3^ The rate measurements indicated that the rate-limiting step is not the reductive half-reaction of FAD by S-nicotine but the oxidative half-reaction of reduced FAD with O_2_. It was argued that an apparent lower *k*_cat_ value might result from the presence of Asn462 in NiAc2, which is substituted with other aromatic residues in other flavin-based amine oxidases.^25^ To this end, site-directed mutagenesis of Asn462 with aromatic residues resulted in a decrease in the rate of the reductive half-reaction and an increase in the rate of the oxidative half-reaction as compared to that with the wild-type enzyme. Based on the crystal structures of variants, it was apparent that the residues of the aromatic cage are important for binding and catalysis.

Computational studies for the hydride transfer mechanism for a number of enzymes provided useful insights into understanding the mechanism as well as the contribution of the active site residues.^12,26−31^ ONIOM calculations involve a hybrid method which is composed of quantum mechanics (QM)- and molecular mechanics (MM)-generated invaluable mechanistic information for a variety of enzymatic systems.^32−37^ With this method, the substrate and active site residues are treated with density functional theory (DFT) functionals, whereas a model region around the active site is considered with MM force fields. In this regard, the protein environment around the active site as well as chemical interactions with the surrounding residues is taken into consideration.

In this study, the hydride transfer mechanism for NiAc2 was evaluated with ONIOM methods using the available crystal structure of the enzyme complexed with S-nicotine. The hydride transfer process was studied through the model enzyme–reactant complex, the transition states (TSs), and the model enzyme–product complex. Based on these models, the role of active site residues was highlighted, and the energetics of the hydride transfer was evaluated by calculating the activation energy. To the best of our knowledge, there is no computational study reported on the mechanism of NiAc2.

## 2 Computational Details and Methodology

In this study, computational models involving the ONIOM method were formulated to investigate the hydride transfer mechanism for NiAc2. The two-layer ONIOM^33^ method used DFT functionals in the QM layer, including CAM-B3LYP,^38^ M06-2X,^39^ and ωB97XD,^40^ and the AMBER force field^41^ in the MM region using Gaussian 09 package.^42^ The B3LYP functional was used due to its complementary computational results for enzyme reactions; the CAM-B3LYP functional includes both the hybrid quality of B3LYP and the long-range correction.^32,43^ The M06-2X functional generated better results than B3LYP for the main-group chemistry.^44^ ωB97XD includes empirical dispersion with long-range corrections. The restrained electrostatic potential charges for each atom belonging to FAD and S-nicotine were calculated with the HF/6-31G(d) method, and MM parameters were obtained with the antechamber option in AMBER 16.^45,46^ For standard residues, Amber 94 MM charges were used. Only the mechanical embedding option was included in the ONIOM calculations. During the optimization processes, no coordinates were frozen in either QM or MM regions.

The geometries of the reactant complexes (RCs), product complexes (PCs), and TSs were optimized in the gas phase using the 6-31G basis set. The geometries obtained with the 6-31G basis set were further optimized with a larger basis set, 6-31G(d,p). TS structures were validated with one negative eigenvalue, and RC and PC structures were without any negative eigenvalues. Frequency calculations were done at 25 °C and 1 atm. TS structures were validated through intrinsic reaction coordinate (IRC) calculations.^47^ TS structure candidates were first identified by potential energy surface (PES) scans by scanning the bond coordinates that are forming or breaking, and the maximum energy points in the scans were subjected to TS optimization using the Berny algorithm.^48^

**Figure 3:**
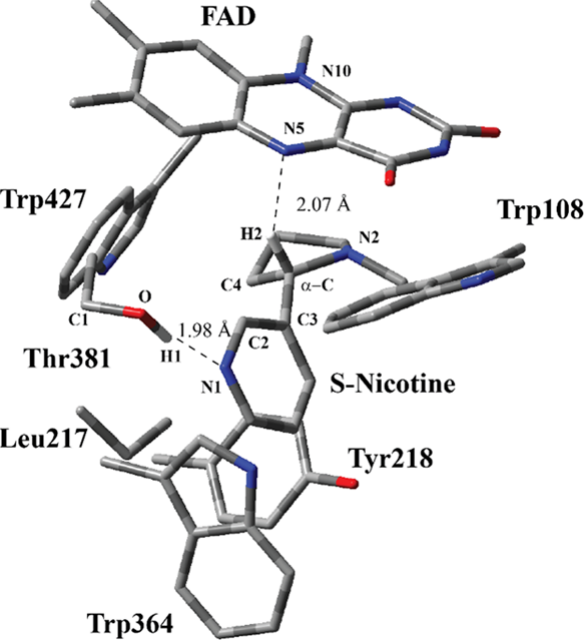
Structure of the optimized RC including FAD, S-nicotine, and six catalytically important residues in the QM region belonging to model M1 (entry #1 in Table 1) obtained with ONIOM(M06-2X/6-31G(d,p):Amber) with tube models excluding H atoms except the ones shown with the ivory color. The distances are given in Å. Numbering of atoms in the isoalloxazine ring is based on the standard pdb numbering of FAD, while numbering for the other residues is arbitrary.

ONIOM calculations employed a model enzyme including FAD, S-nicotine, and the residues around S-nicotine in a diameter of 20 Å. This model was obtained from the crystal structure of the substrate-bound enzyme (PDB accession code: 6C71)^3^ using the VMD program.^49^ It has 1443 atoms and 111 residues including S-nicotine, FAD, and six water molecules. Acetyl and *N*-methyl groups were attached to the *N*-terminal and C-terminal residues on the peripheries. In this way, the electrostatic environment around the active site is maintained. The charges of residues with ionizable groups were determined according to the physiological pH. The total charge of ONIOM models was 0. In the ONIOM model system, the QM region included FAD, S-nicotine, Trp108, Trp364, Trp427, Thr381, Leu217, and Tyr218, while the rest of the residues were placed in the MM region. The QM region included 143 atoms together with a total charge of 0. FAD, Trp108, Trp364, Trp427, Thr381, Leu217, and Tyr218 were split into QM and MM regions, and Figures 2 and 3 show the QM region of these residues.

The model structure obtained from the crystal structure (Figure 2) was optimized using the M06-2X functional with the 6-31G basis set to obtain an initial model enzyme–substrate–cofactor complex. Using this structure, a PES scan was performed on the bond coordinate between the H atom connected to the α-carbon of S-nicotine and the N atom at the N5 position of the FAD ring. The distance was increased in a series of steps to be able to locate the TS structure corresponding to the hydride transfer from S-nicotine to FAD. The hydride transfer process is accompanied with the breaking of the α-C–H bond in S-nicotine and the formation of the N–H bond in FAD (Figure 1). The highest-energy-point geometry in the PES scan was subjected to TS optimization. The optimized structures of RC—which is a reactive model enzyme–substrate complex—and PC were located using appropriate uphill and downhill geometries in the PES scan. These structures were confirmed with the IRC calculations (see the figures in the Supporting Information).

## 3 Results and Discussion

The first step of the computational study was to formulate a model system in which the hydride transfer process can be studied along with the contribution of the catalytically important residues. In this regard, the available crystal structure for the enzyme–substrate complex provides important clues related to the interactions among FAD, S-nicotine, and other residues (Figure 2). The most prominent interaction is the possible H-bonding interaction between Thr381 and S-nicotine. Second, three tryptophane residues including Trp108, Trp364, and Trp427, Tyr218, and the isoalloxazine ring in FAD form a hydrophobic aromatic cage around S-nicotine. In addition to that, Leu217 is poised to have a hydrophobic interaction with S-nicotine and other residues. Asn462, Glu249, and Thr250 are near S-nicotine without any visible direct interactions. It is necessary for S-nicotine to approach the isoalloxazine ring.

### 3.1 ONIOM Model Systems for the Hydride Transfer Mechanism

#### 3.1.1 Reactant Complex

In ONIOM calculations, FAD, S-nicotine, Trp108, Trp364, Trp427, Tyr218, Thr381, and Leu 217 were placed in the QM region, while others were kept in the MM region. The initial structure obtained from the crystal structure (Figure 2) was optimized using the ONIOM(M06-2X/6-31G:Amber) method, followed by a PES scan to locate the RC, TS, and PC. The resultant RC, TS, and PC geometries obtained through the 6-31G basis set were further optimized using a larger basis set, 6-31G(d,p) (Table 1).

**Table 1:** Energy Profile for the Hydride Transfer Process for Different ONIOM (Entries 1–5) and DFT (Entry 6) Model Systems Using CAM-B3LYP, M06-2X, and ωB97XD Functionals in the QM Region^a^

entry #	functional	model system		*E*a_f_	*E*a_r_	imaginary frequency (i)
1	M06-2X	M1	6-31G(d,p)	22.76	17.13	–950.20
2	M06-2X	M2	6-31G	16.43	19.62	–803.14
3	CAM-B3LYP	M3	6-31G	20.26	18.12	–909.49
4	ωB97XD	M4	6-31G	17.97	18.37	–896.77
5	M06-2X	M5-noresid	6-31G	20.50	17.60	–803.14
6	M06-2X	M6-DFT	6-31G(d,p)	27.92	23.07	–1035.50

a *E*_af_: activation energy for the forward reaction in kcal/mol, *E*_ar_: activation energy for the reverse reaction in kcal/mol; noresid denotes ONIOM calculations with FAD and S-nicotine in the QM region and all other residues in the MM region; and DFT denotes calculations with FAD, S-nicotine, and six catalytically important residues only with the M06-2X functional without MM calculations.

The optimized geometry of the RC (Figure 3) shows several important differences from the structure of the active site in the crystal structure (Figure 2). S-nicotine moved almost 1 Å closer toward the FAD ring. Second, the nitrogen atom, N2 in Figure 3, in the pyrrolidine ring experienced a pyramidal inversion, which brings it closer to the FAD ring. A similar finding was reported experimentally in a study in which the crystal structures of 6HLNO in different reaction states were resolved.^50^ In addition, a computational study reported that the hydride transfer process requires the inversion process for 6HLNO.^12^ The pyrrolidine ring of S-nicotine is beneath the isoalloxazine ring. The H atom at α-C, H2, is 2.07 Å away from the N5 atom in FAD. The dihedral angle between N atoms of N5 and N10 positions at isoalloxazine is 29.9° for the crystal structure (Figure 2), whereas this value drops to 14.6° for the RC (Figure 3) based on ONIOM calculations. Bending of isoalloxazine is still prominent, albeit less as compared to that of the crystal structure. The relative positions of Trp108, Trp364, Trp427, Tyr218, and Leu 217 in reference to FAD and S-nicotine did not change appreciably as compared to that in the crystal structure. A similar aromatic cage surrounds S-nicotine in addition to a close H-bonding interaction between S-nicotine and Thr381. CAM-B3LYP and ωB97XD functionals produced similar RC, TS, and PC structures to that by the M06-2X functional.

#### 3.1.2 Transition State

**Figure 4:**
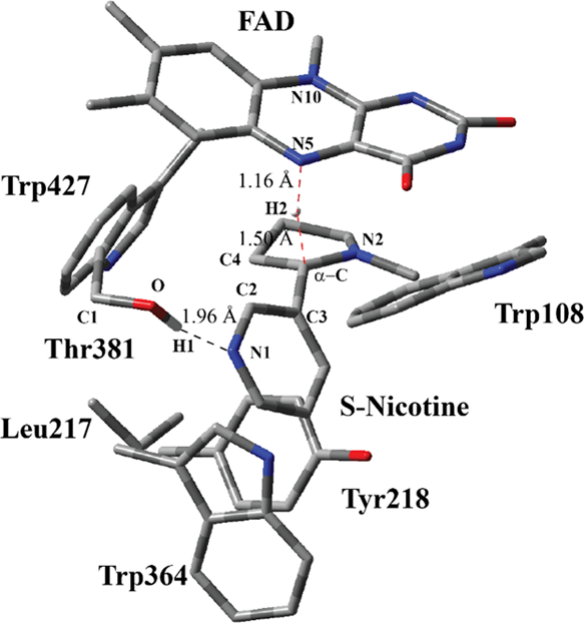
Structure of the optimized TS including FAD, S-nicotine, and six catalytically important residues in the QM region belonging to model M1 (entry #1 in Table 1) obtained with ONIOM(M06-2X/6-31G(d,p):Amber) with tube models excluding H atoms except the ones shown with the ivory color. The distances are given in Å. Numbering of atoms in the isoalloxazine ring is based on the standard pdb numbering of FAD, while numbering for the other residues is arbitrary.

A relaxed PES scan was carried out using the bond coordinate between the H atom connected to the α-C at S-nicotine and the N atom at N5 positions of isoalloxazine for the crystal structure. The TS structure was obtained by optimizing the highest-energy-point geometry in the PES scan. In the optimized TS structure, Thr381 has H-bonding interaction with pyridyl N at S-nicotine similar to that of the RC (Figure 4). The bond between the H atom (H2) and α-C in S-nicotine is almost broken, and the H2 atom in the form of a hydride anion moves toward N5 at the isoalloxazine ring. In addition, the bond distance between α-C and the N2 atom in S-nicotine decreases from 1.48 Å (RC in Figure 3) to 1.37 Å (TS in Figure 4), while α-C starts assuming a more sp^2^ character. The planes of pyrrolidone and pyridyl rings are almost perpendicular in the RC, with a dihedral angle of 95° between the C atoms of pyrrolidone and pyridyl rings, C2–C4, while this angle reduces to 65° in the TS structure. It might be easier for the pyrrolidine ring to move toward the isoalloxazine ring and rotate along the α-C–C3 bond as compared to the pyridyl ring since the pyridyl ring has H-bonding interactions with Thr381. As the hydride leaves α-C, N2 rehybridizes itself to a positively charged sp^2^ nitrogen. Bending along the N5–N10 axis for the isoalloxazine ring in the TS structure is similar to that in the RC with a dihedral angle of 13°.

The activation energy (*E*_af_), which is the absolute energy difference between the TS and RC, for the hydride transfer was calculated to be 16.43 kcal/mol with the M062X functional with the 6-31G basis set in the QM region (*E*_af_, at entry #2 in Table 1). This value increases to 20.26 kcal/mol with CAM-B3LYP and 17.97 kcal/mol with the ωB97XD functional. The estimated activation barriers do not differ significantly from each other. With a higher basis set with the M062X functional 6-31G(d,p), it increases to 22.76 kcal/mol. For the same functional and basis set, the activation energy in terms of the Gibbs free energy change was calculated to be 23.25 kcal/mol, which shows minimal contribution of entropy to the activation barrier. This finding is also in agreement with the observation of no major rearrangement of the active site during the transfer of the hydride ion from the RC to TS. Based on the reported *k*_red_ value of the native enzyme, which is ca. 260 s^–1^,^25^ the M062X functional with the 6-31G basis set estimates a better activation barrier, which is 16.43 kcal/mol (*E*_af_, at entry #2 in Table 1). Using the Arrhenius equation, a rate value of 260 s^–1^ corresponds to an activation barrier of ca. 14.2 kcal/mol at 25 °C and 1 atm. In addition, the activation energies calculated with ωB97XD and M062X functionals are closer to reported activation barriers for MAO and other oxidases.^4,6,51^ The activation barrier for the system (model M5, entry #5 in Table 1) having FAD and S-nicotine in the QM region and all other residues in the MM region resulted in a 4 kcal/mol higher activation barrier than that of the model system (M2, entry #2 in Table 1). This suggests that MM calculations coupled with the minimal QM region could reasonably portray energetics and noncovalent chemical interactions between the residues, substrate, and cofactor. H-bonding interaction between Thr381 and pyridyl N was estimated similarly, even though the former is placed in the MM region and the latter in the QM region (Figure S1). In addition, the aromatic cage placed in the MM region was also predicted to be close to the geometry predicted by QM calculations.

There are several reported computational studies on the hydride transfer mechanism of the MAO family enzymes.^4,5,12,19^ Akyüz and Erdem reported a QM–MM study investigating the oxidation mechanism of benzylamine or phenethylamine by MAO-A and MAO-B.^4^ It was reported that a two-step hydride transfer process might work for MAO-B, whereas MAO-A might undergo a one-step direct hydride transfer process. They reported activation barriers for the hydride transfer process within a range of 23–29 kcal/mol. Cakir et al. reported an ONIOM study of the hydride transfer mechanism using the QM–QM approach to study the oxidation of serotonin by MAO. They reported an activation barrier close to the experimental value. Recently, another study reported DFT and ONIOM investigation of the direct hydride transfer mechanism for LHNO, which is structurally a similar enzyme to NiAc2.^12^ Based on the computational models, it was found that three active site residues as well as a water molecule have roles in the binding and catalysis through a series of H-bonding interactions. The conformation of FAD for the substrate-bound enzyme was reported as bent, which is a similar finding in our study. Besides, the calculated activation barrier was predicted to be close to the experimental value. Interestingly, the active site was found to be reorganized considerably before the hydride transfer step with respect to the crystal structure of the substrate-bound enzyme.

#### 3.1.3 Product Complex

**Figure 5:**
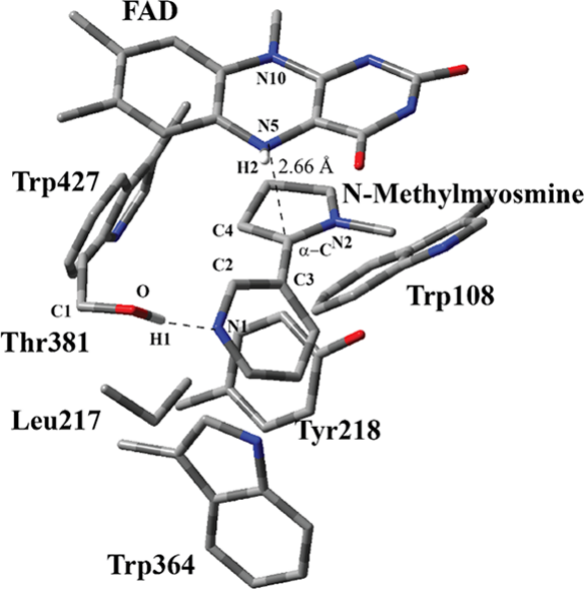
Structure of the optimized PC including FAD, *N*-methylmyosmine, and six catalytically important residues in the QM region belonging to model M1 (entry #1 in Table 1) obtained with ONIOM(M06-2X/6-31G(d,p):Amber) with tube models excluding H atoms except the ones shown with the ivory color. The distances are given in Å. Numbering of atoms in the isoalloxazine ring is based on the standard pdb numbering of FAD, while numbering for the other residues is arbitrary.

The PC was optimized using a proper downhill energy point in the PES scan. The resultant PC geometry (Figure 5) is a complex of the reduced isoalloxazine and the iminium cation surrounded by the active site residues. The hydride ion is completely attached to N5 in FAD, producing reduced isoalloxazine and the iminium cation, *N*-methylmyosmine. The α-C and N2 bond distance decreased, and these atoms assumed sp^2^ characters. The dihedral angle for C2–C4 switched from 65° (TS in Figure 4) to 45° (PC), indicating approximately 21° rotation of the pyrrolidine ring with respect to the pyridyl ring. As a result, the pyrrolidine ring is roughly parallel to the isoalloxazine ring. As in the case of the RC and TS, H bonding interaction of pyridyl N with Thr381 and the aromatic cage around *N*-methylmyosmine exist in the PC. Based on the RC, TS, and PC, it could be concluded that the substrate moves in the aromatic cage, gets closer to the isoalloxazine ring, and undergoes the hydride transfer process with minimal rearrangement of residues in the active site. Bending along the N5–N10 axis for isoalloxazine in the PC decreased from 13° (Figure 4) to 9° (Figure 5).

The activation energy for the reverse hydride transfer process (*E*_ar_), which is the absolute energy difference between the TS and PC, was estimated similarly to be around 17–19 kcal/mol with the three functionals.

### 3.2 Pure DFT Calculations

In addition to ONIOM calculations, the hydride transfer mechanism was studied with pure DFT calculations using S-nicotine, isoalloxazine, and six catalytically important active site residues. The model systems were mainly composed of the atoms of the QM region for ONIOM calculations. In order to mimic the active site environment in the crystal structure, the coordinates of α-C atoms in six catalytically important residues were frozen initially. However, most of the calculations failed to converge in the presence of these constraints. For that reason, no coordinate constraints were used for pure DFT calculations. PES scans using the RC structure obtained with ONIOM calculations were used to locate RC, TS, and PC structures (Figures S2–S4) for the hydride transfer process for pure DFT calculation using the M06-2X functional. The single-point energy calculation was done on gas-phase optimization using the conductor-like polarizable continuum model^52^ with a dielectric constant of 4.0^53^ to represent the model protein environment.

Some fundamental differences were observed for pure DFT calculations as compared to ONIOM calculations. The resulting RC structure (Figure S2) was very different from the RC structure (Figure 3) obtained with the ONIOM method. First, the isoalloxazine ring was estimated to be planar with DFT calculation. Second, the arrangement of six catalytically important residues around S-nicotine is quite different from the RC structure obtained with the ONIOM method. It has to be stressed here that the geometry of the active site obtained with the ONIOM method (Figure 3) was estimated to be similar to the crystal structure (Figure 2). Finally, the conformation of S-nicotine is calculated differently with the DFT method. Furthermore, DFT calculations resulted in considerable rearrangement of residues for the TS structure (Figure S3). As the hydride ion moves from S-nicotine, residues move significantly around S-nicotine with respect to the RC structure (Figure S2). It has to be considered that ONIOM calculations did not estimate any major rearrangement of residues during the transfer of the hydride ion. From the TS to PC (Figure S4), the rearrangement of residues is not significant for DFT calculations. The isoalloxazine ring starts bending slightly during the TS, and for the PC, it has a 4° dihedral angle for N5–N10. This is an expected phenomenon considering the N5 transition from the sp^2^ to sp^3^ hybridization state. In terms of energetics, the DFT calculations produced an activation energy of 27.92 kcal/mol (model M6, entry #6 in Table 1), which is more than any of the values predicted by ONIOM calculations. As a common result, H-bonding interaction between Thr381 and pyridyl N of S-nicotine was estimated in a similar fashion by both ONIOM and pure DFT calculations.

ONIOM methods place a real protein environment as the MM region around the active site, which is treated with QM methods. For that reason, the presence of a real steric and electronic environment around the substrate and cofactor may indeed result in more correct model enzyme–substrate complexes, TS structures, and model enzyme–product complexes. In addition, the energetics of catalysis may be represented with a more realistic approach by ONIOM calculations.

## 4 Conclusions

In this study, ONIOM calculations based on the AMBER force field and DFT functionals and pure DFT calculations were used to investigate the hydride transfer mechanism. The geometries and energetics of the oxidation of S-nicotine into *N*-methylmyosmine catalyzed by NicA2 were studied. It was found that ONIOM methods produced model systems that estimate reasonable reactant and product complexes together with TS structures. With these models, the role of active site residues was highlighted as the hydride ion transfers from S-nicotine to the isoalloxazine ring. It was found that before, after, and during the hydride transfer process, four active site residues form an aromatic cage around S-nicotine, while a threonine residue has a close H-bonding interaction with the pyridyl ring. Furthermore, ONIOM calculations estimated a bent isoalloxazine conformation, which is in agreement with the crystal structure and also with similar FAD-based enzymes. In addition, the geometry of the active site together with the substrate was predicted differently by ONIOM and DFT calculations. ONIOM methods yielded a similar geometry to the crystal structure. The calculated activation barrier for the hydride transfer process was estimated to be close to the reported *k*_red_ value^25^ for the native enzyme by the ONIOM(M06-2X/6-31G) method. Since the oxidation of flavin was found to be the rate-limiting step for the overall reaction,^25^ future mechanistic studies may be allocated to the oxidation of reduced isoalloxazine by a potential electron acceptor such as molecular oxygen. Our results based on ONIOM models provide invaluable insights into understanding the oxidation of S-nicotine by NicA2 and provide a general mechanistic approach for amine oxidases.

## Supporting Information Available

Structures obtained with model systems; Gibbs free energy, enthalpy, and zero-point-corrected electronic energies of ONIOM calculations with the 6-31G(d,p) basis set with the M06-2X functional and pure DFT calculations for the RC, TS, and PC; and Cartesian coordinates of all species in the pure DFT model (PDF)
Gaussian input files for ONIOM calculations with the 6-31G(d,p) basis set with the M06-2X functional for the RC, TS, and PC (ZIP)
