# Supplementary material for: Computational Analysis of the Nicotine Oxidoreductase Mechanism by the ONIOM Method
Source: ACS Omega. 2021 Aug 18;6(34):22422–8. doi: 10.1021/acsomega.1c03357 (PMC8412962; doi:10.1021/acsomega.1c03357)
Supplement: Supplementary file 1 — ao1c03357_si_001.pdf [file ao1c03357_si_001.pdf]

Supporting Information

Computational Analysis of Nicotine Oxidoreductase (NicA2) Mechanism by ONIOM Method

Author(s): Ibrahim Yildiz[†*]

[†]Khalifa University, Chemistry Department, PO Box 127788, Abu Dhabi, UAE Tel: +971 (0)2 401 8208

*E-mail: ibrahim.yildiz@ku.ac.ae

Supporting Figures

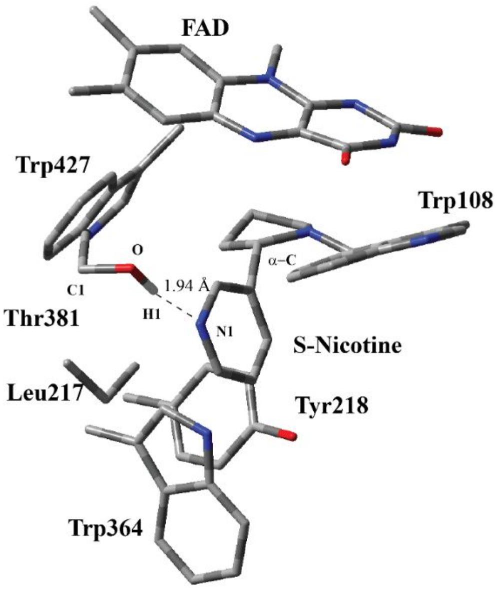

Figure S1. The structure of optimized RC including FAD and S-Nicotine in QM region while six catalytically important residues in MM region belonging to model M5-noresid (Entry #5 in Table 1) obtained with ONIOM(M06-2X/6-31G:Amber) with tube models excluding H atoms except the ones shown with ivory color.

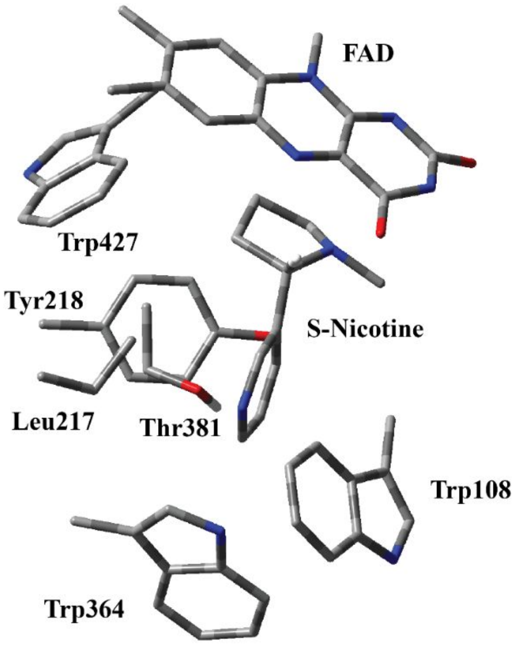

Figure S2. The structure of optimized RC including FAD, S-Nicotine, and six catalytically important residues belonging to model M6-DFT (Entry #6 in Table 1) obtained with M06-2X/6-31G(d,p) with tube models excluding H atoms except the ones shown with ivory color.

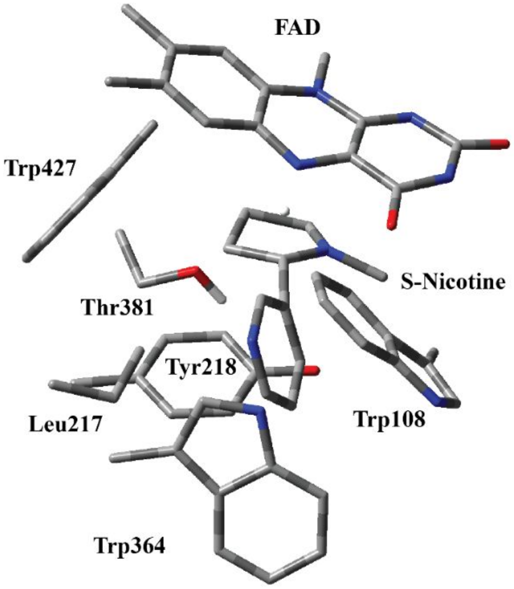

Figure S3. The structure of optimized TS including FAD, S-Nicotine, and six catalytically important residues belonging to model M6-DFT (Entry #6 in Table 1) obtained with M06-2X/6-31G9(d,p) with tube models excluding H atoms except the ones shown with ivory color.

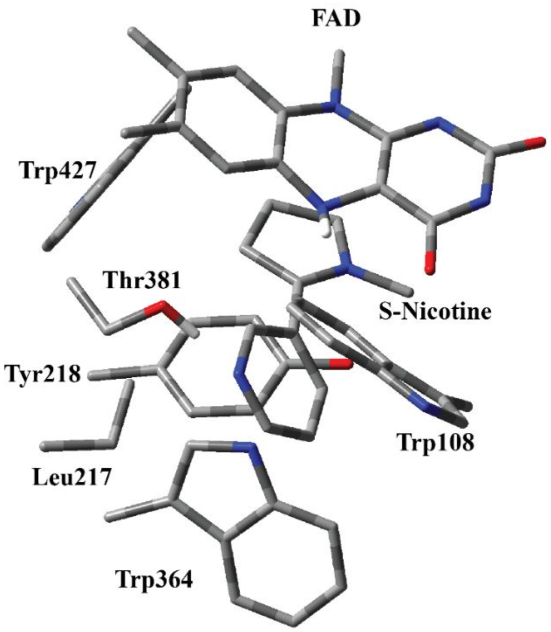

Figure S4. The structure of optimized PC including FAD, S-Nicotine, and six catalytically important residues belonging to model M6-DFT (Entry #6 in Table 1) obtained with M06-2X/6-31G9(d,p) with tube models excluding H atoms except the ones shown with ivory color.

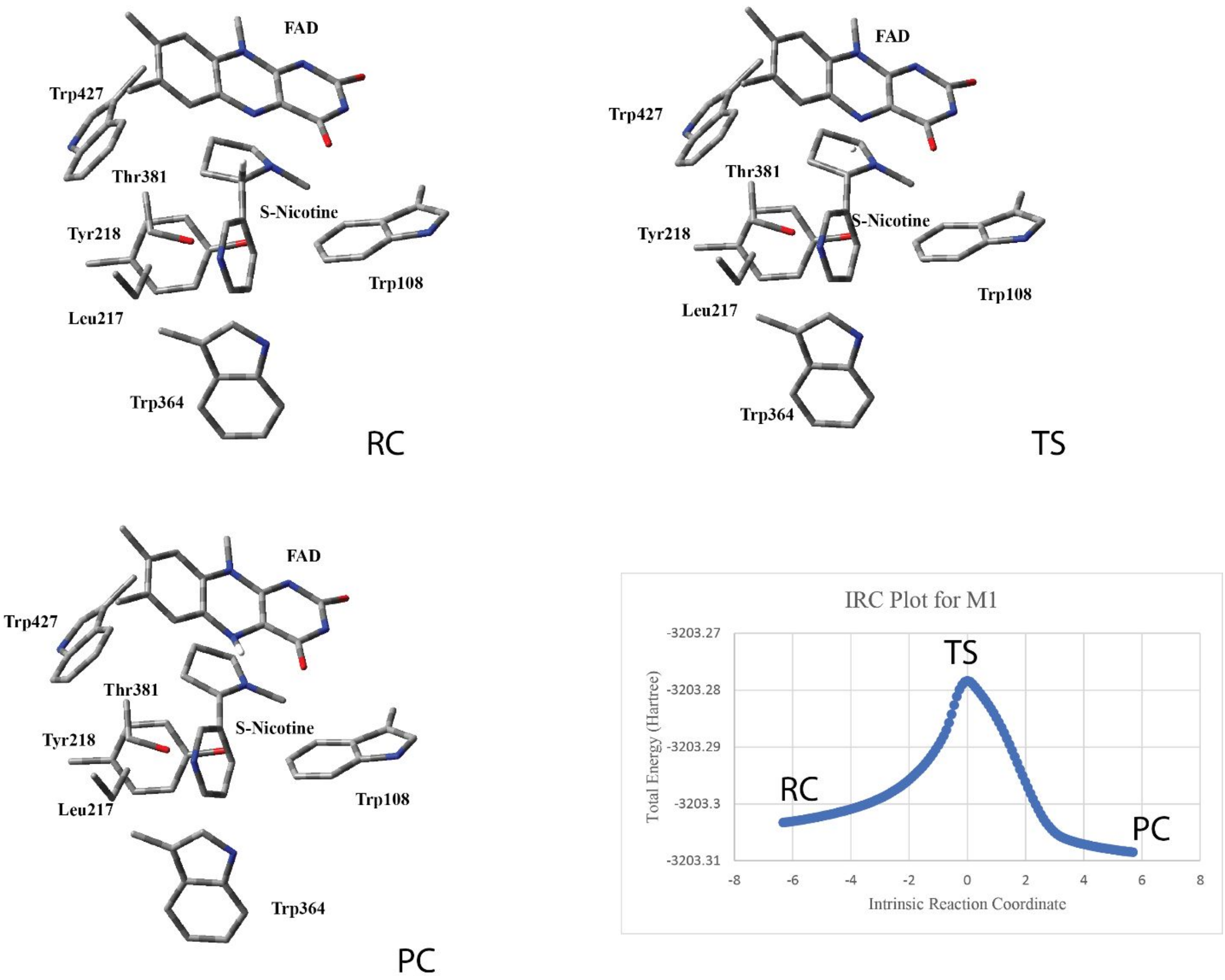

Figure S5. The structures of potential RC, TS, and PC including FAD, S-Nicotine, and six catalytically important residues along IRC coordinate and the IRC plot belonging to model M1 (Entry #1 in Table 1) obtained with ONIOM(M06-2X/6-31G(d,p):AMBER) with tube models excluding H atoms except the one shown with ivory color. (RC and PC structures are taken from the IRC path at the end points of x axis)

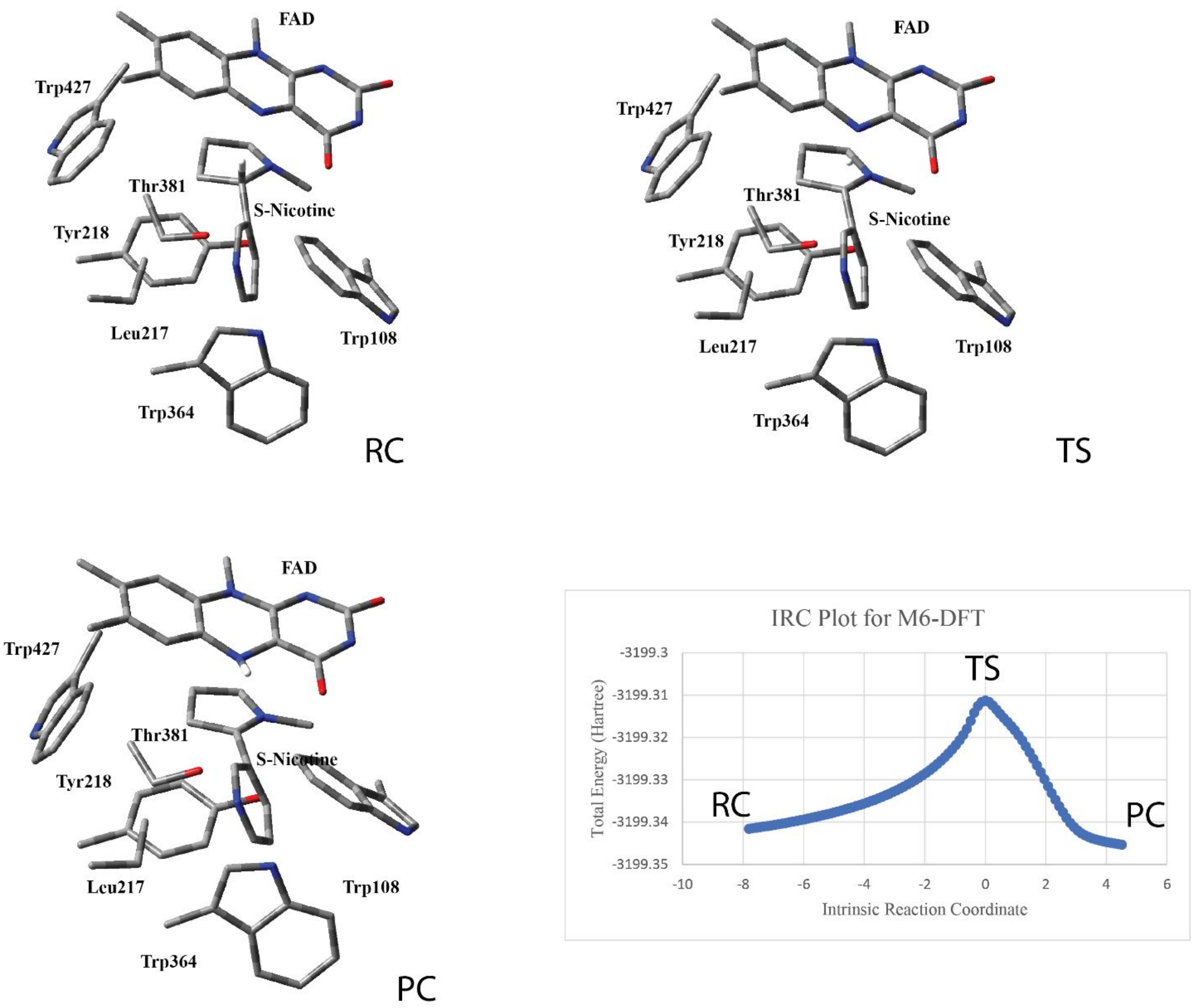

Figure S6. The structures of potential RC, TS, and PC including FAD, S-Nicotine, and six catalytically important residues along IRC coordinate and the IRC plot belonging to model M6-DFT (Entry #6 in Table 1) obtained with M06-2X/6-31G(d,p) with tube models excluding H atoms except the one shown with ivory color. (RC and PC structures are taken from the IRC path at the end points of x axis)

Supporting Tables

Table S1 Absolute Energies of Reactant Complex, Product Complex, and Transition State for the Hydride Transfer Process for Model M1 (Entry #1 in Table 1) with M062X functional with 6-31G(d,p) basis set (ZPE: Zero-point energy correction, H: Enthalpy, G: Gibbs free energy)

Species	E+ZPE (au)	H (au)	G (au)	Imaginary Frequency (*i*)
**RC**	-3192.1354	-3191.3686	-3192.9928	
**TS**	-3192.1026	-3191.3368	-3192.9557	-914.46
**PC**	-3192.1245	-3191.3581	-3192.9774	

Table S2. Absolute Energies of Reactant Complex, Product Complex, and Transition State for the Hydride Transfer Process for Model M6-DFT (Entry #6 in Table 1) with M062X functional with 6-31G(d,p) basis set (ZPE: Zero-point energy correction, H: Enthalpy, G: Gibbs free energy)

Species	E+ZPE (au)	H (au)	G (au)	Imaginary Frequency (*i*)
**RC**	-3198.9793	-3198.9017	-3199.0976	
**TS**	-3198.9379	-3198.8609	-3199.0537	-1035.50
**PC**	-3198.9676	-3198.8900	-3199.0847	

Cartesian Coordinates Corresponding to the Optimized Geometries of All Species with DFT Calculations

Entry #6 in Table S1

<u>**RC**</u>

C	3.38144800	3.41470100	-1.15473000
C	4.77775700	3.29078900	-0.63068000
C	5.94257700	3.50598200	-1.30962800
C	5.16004200	2.90253300	0.70349800
N	7.02901500	3.26690900	-0.48796700
C	6.57560300	2.89925200	0.75641400

C	4.43659500	2.56211400	1.85844900
C	7.27911700	2.55855900	1.91772300
C	5.12584000	2.22060300	3.01172100
C	6.53596400	2.22131600	3.03791500
C	1.43540800	-3.54479900	0.53067800
C	0.34697900	-2.51696500	0.24284000
C	1.15149400	-4.35766700	1.79123300
C	-1.03329800	-5.40106300	-1.50089400
C	-0.87482400	-4.18457100	-2.37428200
C	-1.93344000	-3.29815400	-2.57777500
C	0.35224500	-3.89179400	-2.97646600
C	-1.77439700	-2.15673300	-3.36049200
C	0.52980400	-2.75249500	-3.75051100
C	-0.54086300	-1.88263900	-3.94671500
O	-0.32061200	-0.77619900	-4.72100700
C	4.85861300	-4.05462700	1.60923900
C	5.35157500	-2.70952200	1.17700100
C	5.16195100	-1.51235500	1.81657100
C	6.15282100	-2.41809800	0.01695700
N	5.79520900	-0.50175400	1.13312100
C	6.40517200	-1.02261200	0.02256900
C	6.66816500	-3.19644000	-1.03148700
C	7.12728400	-0.38936200	-0.99499100
C	7.39525000	-2.57728900	-2.03323700
C	7.61741900	-1.18530300	-2.01606100
C	1.99790100	-1.01066400	3.31645800
O	2.75223300	0.17950300	3.20387900
C	0.53270900	-0.71626800	3.61197900

C	-6.16316600	-1.65638400	-1.30721300
C	-5.36226100	-2.83314200	-0.84564800
C	-5.43996400	-4.12602600	-1.28218100
C	-4.38591600	-2.82620000	0.21235300
N	-4.57094400	-4.92347500	-0.56175200
C	-3.91917700	-4.15164800	0.37214200
C	-3.87328800	-1.81478700	1.03671200
C	-2.97322700	-4.49478800	1.34477900
C	-2.93622400	-2.14805200	2.00013700
C	-2.49743700	-3.47749100	2.15625300
N	2.66733800	0.21505700	0.41254200
C	1.48769400	0.83962500	0.39919100
C	0.65521300	0.92260100	-0.71507800
C	1.12291600	0.35130100	-1.90044200
C	2.37139900	-0.26117200	-1.91016100
C	3.10202000	-0.33194800	-0.72576500
N	-1.29683900	2.29581900	-1.61749400
C	-0.73563400	1.48578600	-0.52516900
C	-1.75155800	0.32522600	-0.35205000
C	-2.88797000	0.60970600	-1.34438400
C	-2.21315600	1.48132400	-2.40245800
C	-0.38869200	3.14502500	-2.36310400
N	-4.18618800	4.35305300	-1.54718200
C	-3.10213100	5.13483900	-1.90769700
O	-3.00659400	5.67596300	-2.98637500
N	-2.07728100	5.32067600	-0.95761200
C	-1.82182600	4.49968200	0.11655000
O	-0.77055800	4.50087000	0.71753900

C	-2.99063300	3.63576300	0.47561600
N	-2.91699000	2.90564100	1.53398000
C	-3.99127100	2.09953400	1.83602500
C	-3.90605900	1.25361000	2.95461800
C	-4.92783600	0.38744500	3.29088200
C	-4.79582200	-0.54973000	4.45994200
C	-6.08816900	0.36195800	2.47480000
C	-7.17049900	-0.64486700	2.74858100
C	-6.20161900	1.21518500	1.38620800
C	-5.16079900	2.09372900	1.04876300
N	-5.22571000	2.93476400	-0.05243700
C	-4.14011400	3.68228000	-0.43553700
C	-6.41415200	2.92160400	-0.90096500
H	2.73512500	3.93005100	-0.43603600
H	2.93781100	2.43254600	-1.34783100
H	6.08999400	3.81453000	-2.33505600
H	7.99330200	3.39571100	-0.74317800
H	3.35078000	2.55501000	1.84803400
H	4.56592800	1.92142000	3.89061500
H	8.36430000	2.54955000	1.93722600
H	7.05090300	1.93973900	3.95041400
H	2.40436300	-3.03479300	0.62262000
H	0.27798800	-1.77647300	1.04982200
H	-0.63895200	-2.98793100	0.15283500
H	0.55071600	-1.98578800	-0.69197800
H	1.93328400	-5.09775800	1.98529600
H	1.08023200	-3.70483900	2.66953000
H	0.19582000	-4.88853200	1.70276700

H	-0.38806200	-6.21802200	-1.83505300
H	-2.06889300	-5.75241400	-1.50756200
H	-2.90190800	-3.49701500	-2.12321200
H	1.18766100	-4.57398500	-2.83984200
H	-2.61510600	-1.48752400	-3.53084700
H	1.47858800	-2.53469300	-4.22904900
H	-1.15865400	-0.32662600	-4.88167200
H	4.18098800	-3.96114400	2.46347600
H	4.30952300	-4.55696500	0.80442600
H	4.61961500	-1.28387400	2.72454000
H	5.74256300	0.47679400	1.37888100
H	6.49511400	-4.26899200	-1.05464100
H	7.79860600	-3.16765500	-2.84988700
H	7.28256600	0.68574500	-0.97855400
H	8.18209400	-0.72871800	-2.82314000
H	2.08416900	-1.60168400	2.39289000
H	-0.02815600	-1.64570900	3.76580400
H	0.06907500	-0.17174300	2.78203100
H	0.44889700	-0.10236300	4.51289300
H	3.01935000	0.25151500	2.26519000
H	-6.81242800	-1.28897000	-0.50333500
H	-5.51790900	-0.82215400	-1.60365100
H	-6.05795700	-4.55270800	-2.05964000
H	-4.47080200	-5.91893400	-0.66881000
H	-4.20045800	-0.78585100	0.90666000
H	-2.52315600	-1.37201100	2.63954000
H	-2.62525500	-5.51707700	1.46028900
H	-1.75541900	-3.70550900	2.91543900

H	1.17745300	1.28442600	1.34580000
H	0.52129300	0.36174500	-2.80543200
H	2.76478000	-0.70869000	-2.81610200
H	4.05733700	-0.84754400	-0.68309900
H	-3.70498400	1.16077800	-0.87009400
H	-3.30333200	-0.31739200	-1.74879300
H	-1.29982100	5.88427700	-1.28092700
H	-2.98762900	1.29737800	3.53397000
H	-4.82896400	-1.59274900	4.12580400
H	-3.84576200	-0.39278200	4.97474400
H	-5.60322300	-0.40571800	5.18511900
H	-6.78109400	-1.65840200	2.59300100
H	-8.02449800	-0.50156000	2.08366700
H	-7.52451200	-0.58737900	3.78225600
H	-7.09966500	1.17246400	0.78097700
H	-6.52686900	1.93561500	-1.36294900
H	-6.28139300	3.67536300	-1.67162100
H	-0.70590900	2.11037700	0.37772300
H	-2.09341800	0.25269400	0.68302500
H	-1.28213600	-0.62998700	-0.60522600
H	-2.91028700	2.12644300	-2.95108600
H	-1.68122700	0.84592700	-3.13748900
H	-0.96870300	3.83259400	-2.98851000
H	0.20968200	3.73109800	-1.65660500
H	0.30308800	2.59037900	-3.01578200
H	3.36371700	3.97955100	-2.09065600
H	5.68396300	-4.71288900	1.90223900
H	2.41706400	-1.62120600	4.12973900

H	1.52931700	-4.22312200	-0.32800300
H	-0.77092000	-5.17093500	-0.46105200
H	-6.79502800	-1.91675700	-2.15960000
H	-7.29494300	3.14543000	-0.29591200

TS (Transition State)

C	3.68847300	2.94236100	-1.88650900
C	4.90094700	2.81462100	-1.02100600
C	6.21006300	2.96415200	-1.37802800
C	4.90300400	2.50426000	0.38571000
N	7.03271400	2.75850800	-0.28315300
C	6.25316800	2.47509800	0.81309200
C	3.87897100	2.26483500	1.31704600
C	6.60767500	2.19404700	2.13813000
C	4.22516400	1.97499600	2.62846000
C	5.57750100	1.93924200	3.03089300
C	1.19544600	-4.04569200	0.24491500
C	0.07700800	-3.02798000	0.04528000
C	0.89595800	-5.01028400	1.38914800
C	-2.65537300	-5.11686800	-2.11177800
C	-2.31771400	-3.80231200	-2.76386400
C	-3.29705100	-2.85592500	-3.06167900
C	-0.99600600	-3.51736300	-3.12396700
C	-2.97537400	-1.66906000	-3.72191900
C	-0.65719700	-2.33715200	-3.76765200
C	-1.65340500	-1.40904900	-4.07074500
O	-1.26179000	-0.25243900	-4.69425600
C	4.58520100	-4.44811700	1.66825900
C	5.07839400	-3.10291000	1.23547500

C	4.87393200	-1.90185700	1.86367400
C	5.88870100	-2.81425900	0.08079000
N	5.49828400	-0.89037300	1.17390000
C	6.12501200	-1.41586400	0.07413700
C	6.41447000	-3.59661700	-0.95987800
C	6.83819500	-0.78245700	-0.94979100
C	7.13538900	-2.97788800	-1.96611300
C	7.33944600	-1.58281700	-1.96199500
C	1.69804500	-1.74648100	3.22763600
O	2.29101000	-0.46716200	3.14577700
C	0.22735300	-1.62148800	3.59571500
C	-6.80615100	-0.40647100	-0.33366300
C	-6.20548300	-1.77710600	-0.30980400
C	-6.63264200	-2.88981900	-0.97768700
C	-5.08482600	-2.19788600	0.49259500
N	-5.84270500	-3.97657800	-0.64791900
C	-4.89320800	-3.58008200	0.26668400
C	-4.24692600	-1.52895100	1.39598400
C	-3.89521500	-4.30983000	0.92265900
C	-3.24980400	-2.24392300	2.03875500
C	-3.07779700	-3.62228200	1.80427700
N	2.32067600	-0.45642100	0.34950300
C	1.15291200	0.17625400	0.26227100
C	0.47998300	0.38633600	-0.94238300
C	1.09798000	-0.04376000	-2.11878600
C	2.32491200	-0.69166400	-2.03181300
C	2.89057400	-0.89794500	-0.77558500
N	-1.38786700	1.75750200	-1.85071300

C	-0.92625500	0.86456000	-0.93374400
C	-2.01508700	-0.15469300	-0.64605800
C	-3.29731200	0.53253000	-1.13759700
C	-2.80480700	1.56178200	-2.16372300
C	-0.58852500	2.50747900	-2.80407900
N	-2.78256700	5.11103400	-1.04626800
C	-1.67121600	5.70785400	-1.59800200
O	-1.71761000	6.59254500	-2.42906200
N	-0.40573700	5.24619900	-1.16959300
C	-0.16383300	4.15273700	-0.36559800
O	0.95627100	3.66351100	-0.23776800
C	-1.36316300	3.60058000	0.22737000
N	-1.21002000	2.51746000	1.03968800
C	-2.36129700	2.16070500	1.77601800
C	-2.22694000	1.29187600	2.85979300
C	-3.31643700	0.90743600	3.63261500
C	-3.14991400	-0.03298000	4.79628100
C	-4.58799900	1.41543000	3.30009900
C	-5.80132700	0.97762600	4.07599900
C	-4.72748500	2.29722700	2.23249500
C	-3.62399400	2.69304800	1.46368700
N	-3.74884700	3.57743300	0.39183300
C	-2.61621400	4.14180500	-0.17537600
C	-5.06558800	4.06246600	0.00780500
H	2.98525900	3.67170600	-1.47467000
H	3.14952800	1.98894500	-1.94381100
H	6.63472800	3.20276100	-2.34312900
H	8.03496000	2.84152300	-0.28125300

H	2.83749600	2.33060000	1.01047800
H	3.44848800	1.75815900	3.35403400
H	7.64631200	2.16676300	2.45231800
H	5.81629700	1.70461400	4.06312300
H	2.14017100	-3.51748900	0.43581000
H	-0.06092800	-2.41646100	0.94566200
H	-0.88331300	-3.51626300	-0.15975700
H	0.29500600	-2.35840500	-0.79190500
H	1.69278600	-5.74663400	1.52554900
H	0.77736300	-4.46730600	2.33431600
H	-0.03835100	-5.55286300	1.20296600
H	-2.41372000	-5.95397000	-2.77424700
H	-3.71979500	-5.16616900	-1.87297900
H	-4.33030100	-3.04336200	-2.77608600
H	-0.21493500	-4.23923500	-2.89660900
H	-3.75567900	-0.94936500	-3.96119600
H	0.36691500	-2.12781600	-4.05720700
H	-2.03605900	0.20106600	-5.04775500
H	3.85033800	-4.35122200	2.47373300
H	4.10049200	-4.97730200	0.83971700
H	4.32277400	-1.67214100	2.76567200
H	5.44141500	0.09035100	1.41282800
H	6.25421800	-4.67131100	-0.97327400
H	7.54791800	-3.57102600	-2.77613500
H	6.97783700	0.29476500	-0.94599700
H	7.89862300	-1.12656800	-2.77304300
H	1.80637600	-2.27754800	2.27101800
H	-0.24072900	-2.60194900	3.73957500

H	-0.31703500	-1.09403400	2.80400800
H	0.13037400	-1.04670000	4.52184900
H	2.60611400	-0.36344600	2.22637500
H	-7.10328300	-0.09716600	0.67452800
H	-6.09727500	0.34240700	-0.70264200
H	-7.45790300	-3.00190000	-1.66699900
H	-6.01425000	-4.92528300	-0.93628000
H	-4.36829400	-0.46312200	1.57434600
H	-2.58250000	-1.73381300	2.72763200
H	-3.76822700	-5.37420300	0.75058700
H	-2.28368500	-4.15322700	2.32112200
H	0.73404400	0.53527900	1.20300700
H	0.60585200	0.09653900	-3.07792600
H	2.83607000	-1.05004700	-2.91885700
H	3.82514300	-1.43864800	-0.66470200
H	-3.80781000	1.03361500	-0.31301200
H	-3.99026800	-0.19065600	-1.57459200
H	0.39117800	5.67231100	-1.62546400
H	-1.22601800	0.93985100	3.09955300
H	-3.78787200	-0.91690400	4.68443900
H	-2.11471000	-0.37207800	4.87719900
H	-3.42280400	0.44928700	5.74119000
H	-5.96241800	-0.10080900	3.96017800
H	-6.69965500	1.49414400	3.73201600
H	-5.68483900	1.17251500	5.14678800
H	-5.71422200	2.68252200	2.00202200
H	-5.71695600	3.21296400	-0.22229000
H	-4.94274800	4.69555400	-0.86732400

H	-0.91651400	1.61848000	0.32311700
H	-2.05115100	-0.43908700	0.40916400
H	-1.80322700	-1.06837000	-1.22095700
H	-3.33575600	2.52125200	-2.12523000
H	-2.86786900	1.17794500	-3.19142300
H	-1.08508500	3.46082400	-3.00537500
H	0.40189900	2.69886200	-2.38576800
H	-0.50079400	1.94696100	-3.74285800
H	3.96219100	3.23964000	-2.90231700
H	5.40053800	-5.08492200	2.02875600
H	2.21864900	-2.34342500	3.99153900
H	1.34687300	-4.61275900	-0.68279000
H	-2.09328800	-5.25597200	-1.18246900
H	-7.69311400	-0.37541100	-0.97089300
H	-5.51528700	4.65013400	0.81498300

PC (Product Complex)

C	4.44146200	2.87594000	-2.18715000
C	5.22116900	2.74809000	-0.91765600
C	6.53349000	3.04789600	-0.68750300
C	4.70212800	2.25497400	0.33053800
N	6.86835300	2.75764100	0.62500500
C	5.75688800	2.28150500	1.27521400
C	3.43082600	1.81266300	0.72466100
C	5.57235300	1.87912300	2.60517200
C	3.24543800	1.41510100	2.03839000
C	4.30528300	1.44637000	2.96908800
C	0.93935400	-4.44737300	-0.30793100

C	-0.26645100	-3.51748000	-0.20565600
C	0.84893300	-5.62042200	0.66489500
C	-3.49554500	-4.78208000	-2.21104100
C	-3.06111500	-3.46065300	-2.78818200
C	-3.96785000	-2.41891500	-2.98265200
C	-1.74021600	-3.26869700	-3.20755300
C	-3.58370700	-1.23775400	-3.61660800
C	-1.33604700	-2.09138400	-3.82138600
C	-2.26866600	-1.07877700	-4.04484000
O	-1.83182000	0.05073100	-4.68652800
C	4.45598800	-4.76853500	1.03905800
C	4.90928700	-3.36000100	0.81411000
C	4.63998900	-2.26438200	1.59348500
C	5.73318800	-2.88514500	-0.26797700
N	5.23478000	-1.14515900	1.06279300
C	5.91027800	-1.49117200	-0.07801200
C	6.30723500	-3.49378700	-1.39670100
C	6.62044400	-0.69373200	-0.98243000
C	7.01822600	-2.71251500	-2.29006000
C	7.17004800	-1.32532100	-2.08446900
C	1.46718500	-2.63507900	2.97206600
O	1.80121000	-1.25842600	2.95370400
C	0.05316400	-2.82939800	3.49156500
C	-7.05488200	0.35891000	0.07123800
C	-6.52765000	-1.04088100	0.03220300
C	-7.03741300	-2.10330500	-0.65870800
C	-5.38843000	-1.54380300	0.75931200
N	-6.27774800	-3.23525200	-0.42214700

C	-5.26177200	-2.91832600	0.45153500
C	-4.48731700	-0.95910000	1.66263000
C	-4.25035800	-3.71389700	1.00086800
C	-3.48581900	-1.74266700	2.21148200
C	-3.36583000	-3.10576400	1.87647000
N	1.90636600	-1.03030000	0.14440000
C	0.74342900	-0.40522800	0.01581700
C	0.27093200	0.10624600	-1.20080700
C	1.06638100	-0.06832100	-2.33560200
C	2.28287200	-0.72748700	-2.20249400
C	2.66286900	-1.19453800	-0.94826300
N	-1.49372800	1.59616600	-2.05606100
C	-1.08670100	0.65628000	-1.25326300
C	-2.22931400	0.00490800	-0.53795900
C	-3.46246700	0.82965300	-0.95314200
C	-2.95197700	1.79811300	-2.03083600
C	-0.69065400	2.42780100	-2.94708800
N	-2.33485700	5.16791200	-0.76052300
C	-1.38014000	5.78673500	-1.52498300
O	-1.59003600	6.73036700	-2.26954000
N	-0.06673400	5.26705900	-1.44772700
C	0.32916700	4.14559300	-0.74546100
O	1.46734400	3.65215100	-0.87161500
C	-0.69865900	3.58568800	0.04517800
N	-0.44207300	2.37043200	0.73474700
C	-1.28194900	2.11130500	1.82874800
C	-0.89967400	1.26957300	2.86824000
C	-1.77453700	0.93491700	3.90597600

C	-1.30453000	0.04320700	5.02466100
C	-3.07548300	1.44632200	3.88372400
C	-4.07181600	1.05312900	4.94247600
C	-3.45206200	2.31445200	2.85162400
C	-2.57935500	2.66742300	1.82276000
N	-2.96454900	3.52707900	0.77628100
C	-1.96824700	4.14672500	0.00266800
C	-4.30431700	4.08012500	0.78252400
H	3.50286300	3.40915000	-2.00558700
H	4.18681000	1.88499300	-2.58202400
H	7.27470500	3.44927000	-1.36421800
H	7.76471800	2.92762800	1.04824700
H	2.61416500	1.82752300	0.00656300
H	2.27436200	1.06599800	2.37620900
H	6.38775800	1.90066400	3.32122500
H	4.12172500	1.11622300	3.98628400
H	1.85910600	-3.87736100	-0.11792700
H	-0.32841900	-3.05280600	0.78499500
H	-1.20600700	-4.06020600	-0.36285400
H	-0.21919800	-2.71674600	-0.94989300
H	1.70687100	-6.29215300	0.57423500
H	0.81160300	-5.26666800	1.70139500
H	-0.05927700	-6.20566800	0.48441200
H	-3.54261700	-5.54842800	-2.99170000
H	-4.48674400	-4.69737700	-1.76192600
H	-4.99468000	-2.52866300	-2.64130700
H	-1.01320800	-4.06354400	-3.05933700
H	-4.31476800	-0.44995700	-3.78633700

H	-0.31559900	-1.95570300	-4.16340300
H	-2.59057500	0.53580000	-5.03220000
H	3.70892300	-4.81512100	1.83753300
H	4.00083500	-5.18695100	0.13393500
H	4.05715600	-2.18345900	2.50149100
H	5.14748900	-0.21089200	1.43821400
H	6.19010200	-4.56104600	-1.56405100
H	7.46708800	-3.16969100	-3.16611000
H	6.72296200	0.37639600	-0.82415200
H	7.72913500	-0.74037800	-2.80806500
H	1.56446200	-3.04999700	1.95889800
H	-0.25243800	-3.87872800	3.41968300
H	-0.64507700	-2.21514600	2.90993800
H	-0.01860500	-2.52391000	4.53799500
H	2.13560200	-1.08086900	2.05387700
H	-7.29218000	0.65908300	1.09710700
H	-6.32385900	1.07761000	-0.31523100
H	-7.90271100	-2.14865900	-1.30529300
H	-6.51971800	-4.16555300	-0.71979500
H	-4.56492400	0.09271300	1.92792600
H	-2.78263600	-1.29298200	2.90727900
H	-4.16762500	-4.76956200	0.76157300
H	-2.56753000	-3.69351000	2.31943900
H	0.15263100	-0.29889300	0.92614300
H	0.72180000	0.27086400	-3.30779500
H	2.93077300	-0.89072500	-3.05621400
H	3.59329600	-1.73542500	-0.81245900
H	-3.85048300	1.38624300	-0.09898100

H	-4.25290600	0.17829300	-1.33095600
H	0.62650600	5.72646900	-2.02280000
H	0.10682900	0.85314900	2.87992600
H	-1.90669400	-0.87166500	5.09482000
H	-0.26255700	-0.24611100	4.87052700
H	-1.38247400	0.54968800	5.99291300
H	-4.27756500	-0.02377200	4.90423500
H	-5.01956600	1.57893900	4.80707600
H	-3.70218300	1.27501100	5.94912200
H	-4.45584800	2.72534400	2.86670600
H	-5.04281700	3.26940600	0.73514200
H	-4.39902200	4.73121100	-0.08352100
H	0.55351900	2.27904600	0.93342200
H	-2.06430200	0.00150000	0.54514100
H	-2.29486200	-1.04602900	-0.85624400
H	-3.15426700	2.85205700	-1.81440400
H	-3.32776900	1.57203000	-3.03507900
H	-1.14488800	3.42088400	-2.97166900
H	0.32537100	2.51279200	-2.55612900
H	-0.69916000	1.98962700	-3.94810500
H	5.01512900	3.40548500	-2.95182000
H	5.28853700	-5.42397200	1.31688000
H	2.17458400	-3.18392000	3.61292800
H	1.02345900	-4.82923900	-1.33334000
H	-2.80103200	-5.13647900	-1.44372400
H	-7.96523100	0.45355200	-0.52532800
H	-4.49344900	4.66860400	1.68922000
